# Supplementary material for: Neural Activity Associated with Symptoms Change in Depressed Adolescents following Self-Processing Neurofeedback
Source: Brain Sci. 2022 Aug 25;12(9):1128. doi: 10.3390/brainsci12091128 (PMC9496932; doi:10.3390/brainsci12091128)
Supplement: Supplementary file 1 [file brainsci-12-01128-s001.zip › brainsci-1848498-supplementary.pdf]

1. Online steps to warp the AMYHIPP ROI to each individual brain

Generation of subject specific anatomic masks of the bilateral amygdala and hippocampus (AMYHIPP) region of interest (ROI). The single band reference functional image from a pre-feedback multiband EPI series was used as the target functional reference for the coordinate system transformation since the neurofeedback would be generated from subsequent real-time multiband EPI within the same imaging session. The high-resolution structural image of the subject (MPRAGE) was also used as a structural anatomic reference for registration to the MNI reference. An AMYHIPP mask derived via the WFU_PickAtlas tool was transformed from MNI space into subject's functional imaging space using SPM12 modules [40]. The 4-step process was as follows: 1. Alignment of the subject's functional and structural images series. 2. Segmentation and spatial normalization of the structural image to the MNI coordinate space and output of spatial normalization parameters that perform inverse deformation between coordinate spaces. 3. Warping of the ROI from MNI space to subject space using the spatial normalization parameters yielded by step 2. 4. Registration of the warped ROI to match voxel-for-voxel the specific subject's functional image space to allow for real-time masking. Masks were converted from 8bit to 16 bit for compatibility with MURFI. This last step concluded with overlapping of the ROI on both structural and functional images. **Supplemental Figure S. 1.**

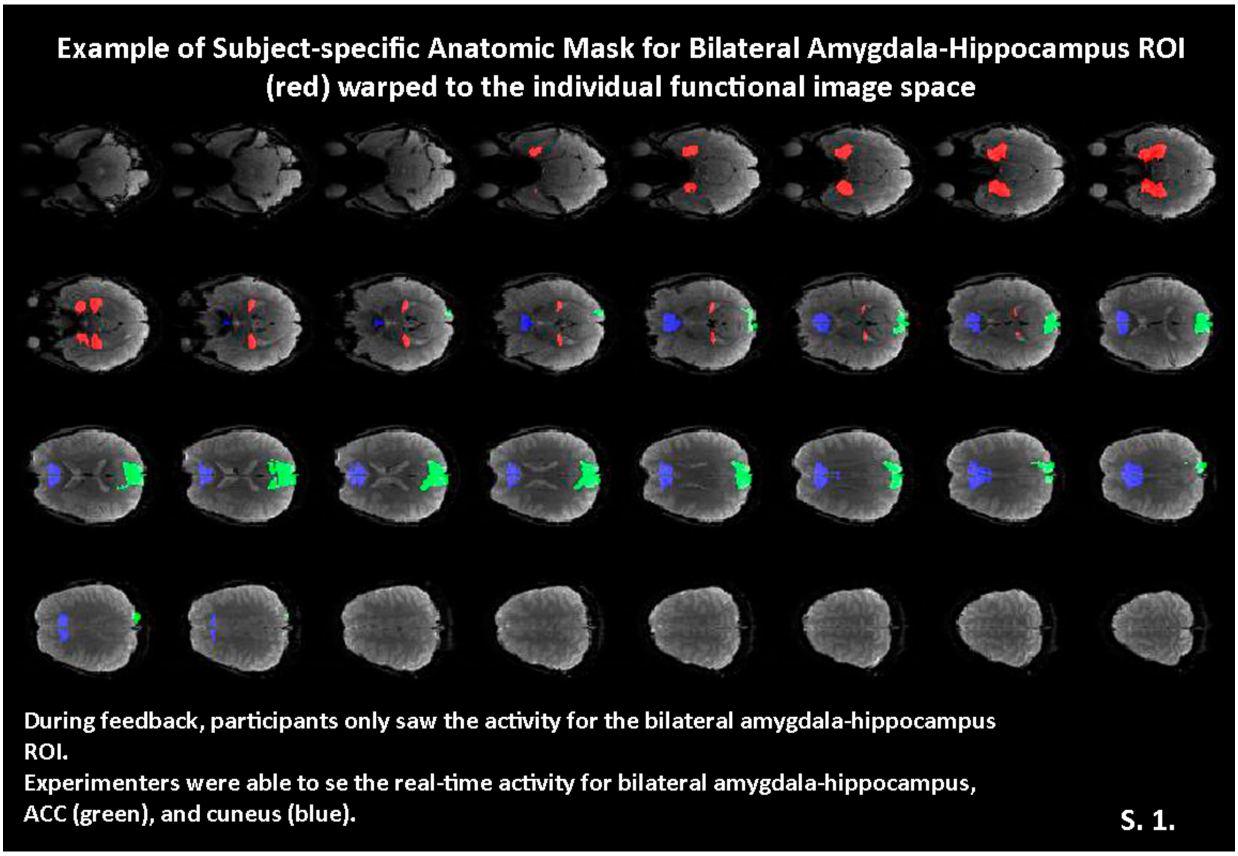

2. **Memory check and happiness before versus NF.** Ratings of happiness before and after the scanning as well as memory recall ratings were analyzed with a repeated measures ANOVA and a one-way ANOVA respectively. A 10-point scale rating measured successful recalling of happy memories during the ESOM_NF task, and happiness before and after NF. There were no significant differences, $F(1,48)=0.397$, $p = 0.53$, for ratings in successful recalling of happy memories between control (M=5.53) and depressed (M=5.18) groups.
   Additionally, analyses of ratings for happiness before and after scanning task showed that healthy control youth tended to have a higher rating in happiness overall at both times. However, the groups did not differ in their ratings before and after the scanning tasks $F(1,50)=2.91$, $p=0.09$.

3. **ESOM Post- versus Pre- Neurofeedback in Whole sample covariate results.**

**Supplemental Table S1:** Areas of Activity Associated with Covariates in a Multiple Regression for the Post- versus Pre- Neurofeedback in Whole sample	Direction of Prediction	Voxels	Hemisphere	MNI Coordinates			T
				x	y	z	
**Depression severity at intake T1 (CDRS total scores)**							
Cerebellum	Positive	577	Left	-30	-68	-42	4.61
Cerebellum		309	Right	32	-62	-40	4.86
Parahippocampus, Fusiform		511	Right	32	-42	-8	4.87
Fusiform, Inferior and Superior Temporal Gyrus		417	Left	-40	-66	-8	4.88
Occipital Gyrus		155	Right	48	-50	-8	4.42
Precuneus, Middle Temporal Gyrus		1116	Right	22	-64	62	4.98
Superior Occipital and Middle Temporal Gyri		182	Left	-28	-82	16	3.61
Posterior Cingulate Cortex, Precuneus		227	Left and Right	0	-50	28	4.08
Precuneus, BA 7 & 40		161	Left	-22	-60	64	4.55
Precentral Gyrus, Inferior Parietal Lobule		421	Right	64	-28	36	4.34
Superior Frontal Gyrus, BA 8 & 6		131	Right	16	28	58	4.72
**IQ (WASI)**							
Precuneus, Cingulate Gyrus, BA 7 & 31	Positive	191	Right	14	-56	34	4.45
Insula, Superior Temporal Gyrus	Negative	285	Left	-46	-2	8	4.13

4. **ESOM Post- versus Pre- Neurofeedback in Whole sample depression and rumination change results.**

**Supplemental Table S2:** Areas of Activity Associated with Depression and Rumination Change in a Multiple Regression for the Post- versus Pre- Neurofeedback contrast	Direction of Prediction	Voxels	Hemisphere	MNI Coordinates			T
				X	y	Z	

**Depression Change linked to Brain Activity during ESOM in all participants Post (Self vs Other) vs Pre (Self vs Other)**							
Middle temporal gyrus (BA 21), superior temporal gyrus (BA 22)	Positive	551	Left	-56	-24	-6	4.67
Cerebellum	Positive	418	Right	20	-80	-26	4.41
Cerebellum	Positive	288	Left	-24	-80	-26	4.38
Middle frontal gyrus	Positive	118	Left	-26	10	36	3.86
Middle temporal gyrus (BA 21), superior temporal gyrus (BA 22)	Positive	160	Right	62	-42	2	3.72
Supramarginal gyrus (BA 40), angular gyrus, superior temporal gyrus,	Positive	226	Left	-36	-54	34	3.71
Putamen, frontal gyrus (BA 47)	Positive	132	Left	-26	10	-10	3.66
**Rumination Change linked to Brain Activity during ESOM in all participants Post (Other vs Self) vs Pre (Other vs Self)**							
Posterior cingulate, cuneus, occipital lobe	Positive	118	Left	-16	-64	4	4.49

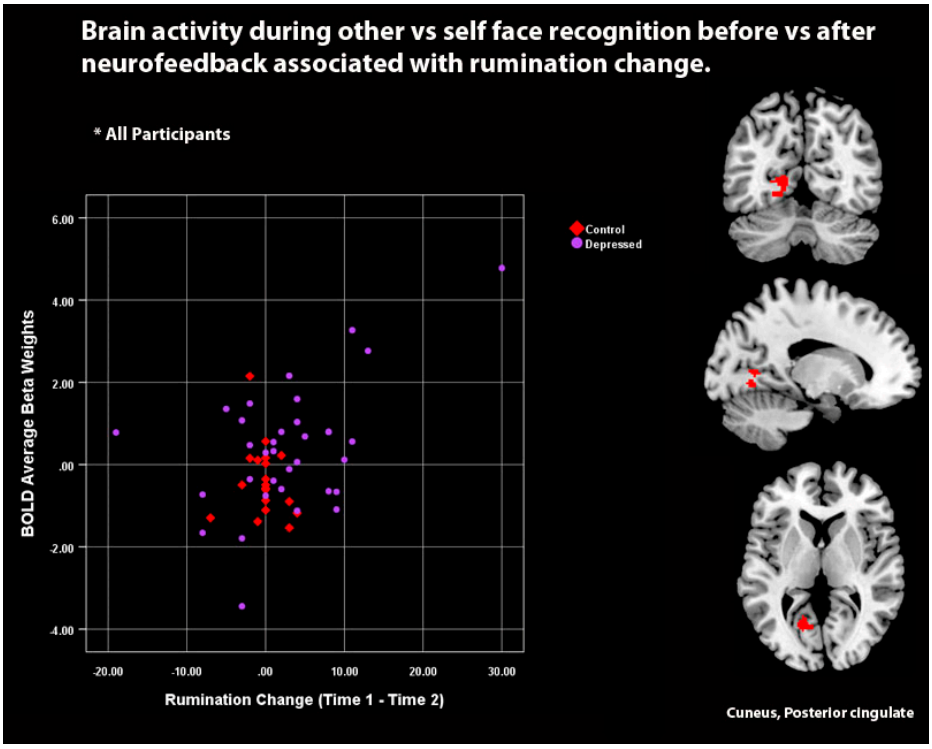

Figure S2. Effect of Depression and Rumination Change Post- versus Pre- Neurofeedback for the Whole Sample, N=53. Higher cuneus and posterior cingulate activity during other vs self-face recognition for ESOM-Post vs ESOM-Pre is associated with Rumination Change in all participants.
